# Diabetic Retinopathy: From Pathogenesis to Treatment

**DOI:** 10.1155/2007/69527

**Published:** 2008-01-16

**Authors:** Subrata Chakrabarti

**Affiliations:** London Health Science Centre, Department of Pathology, The University of Western Ontario, London, ON, Canada N6A 5A5;

In spite of all advances in the understanding of chronic diabetic complications, diabetic retinopathy remains a significant clinical problem. The pathogenetic mechanisms leading to the development of diabetic retinopathy is indeed complex. Multiple interactive mechanisms may come into play leading to cellular damage and adaptive changes leading to the development of this devastating complication of diabetes.

In this focused issue of the journal we have assembled several invited reviews, from well-recognized experts in their fields, as well as original research articles. These reviews provide state-of-the-art knowledge dealing with several mechanisms, all of which contribute to the development of diabetic retinopathy. The reviews include discussion on several pathogenetic mechanisms such as polyol pathway, oxidative stress, cellular signaling, inflammatory changes, and excitatory amino acids as well as pharmacotherapy in diabetic retinopathy. In addition, several excellent original research articles demonstrate novel pathophysologic aspects of this disease process which ranges from population-based studies to mechanistic studies.

It is becoming more and more evident that early diabetic retinopathy is associated with increased production of several inflammatory mediators. Several pharmacological approaches by inhibiting production of inflammatory mediators are effective in preventing early lesions of diabetic retinopathy. Although this concept is relatively new, it has enormous potential as a drug target. Dr. T. S. Kern has discussed the inflammatory process in the pathogenesis of early stages of diabetic retinopathy.

Dr. M. Lorenzi has reviewed polyol pathway activation as a mechanism for diabetic retinopathy. Interest in this pathway stems from the fact that it remains one of the attractive mechanisms to explain several cellular changes in hyperglycemia. With new knowledge of this pathway and availability of novel aldose reduction inhibitors we will probably see new clinical trials in the near future.

Inflammation or other changes like augmented polyol pathway activity leads to oxidative stress in the retina which disrupts the fine balance between formation and elimination of reactive oxygen species. Drs. Kowluru and Chan have emphasized that several metabolic abnormalities which are implicated in diabetic retinopathy are influenced by oxidative stress. Although currently little clinical data is available as to the effectiveness of blocking such pathways for the treatment of diabetic retinopathy, antioxidant treatment remains an attractive future option for pharmacotherapy for diabetic retinopathy. In an accompanied research article this group has also demonstrated that peroxinitrite accumulation and impaired scavenging of mitochondrial superoxide may be an important mechanism in the pathogenesis of retinopathy.

In a research article, Dr. Canning et al. demonstrated the role of advanced glycation end products (AGEs) and galectin 3 with AGE binding properties in the blood retinal barrier breakdown and VEGF upregulation. This research reemphases the importance of such mechanisms in the pathogenesis of early changes in diabetic retinopathy.

Furthermore, Drs. Khan and Chakrabarti discussed a plethora of intracellular signaling mechanisms ranging from second messengers, transcription factors to transcription coactivators that are of importance in functional and structural changes in the microvasculature in diabetic retinopathy.

Dr. Pulido et al. addressed the relationship between diabetic retinopathy and elevated glutamate level. Such elevated glutamate levels may have toxic effects. In an accompanied research article this group also demonstrated a method for detection of glutamate.

Dr. Ross et al. reported results of an interesting clinical study from Alberta, Canada. They have demonstrated that ethnicity does play a role in the development of diabetic retinopathy although the risk factors are similar between various populations.

Finally, Drs. Schwartz and Flynn discussed clinical experience with various pharmacological treatments. As more data is available, the role of such treatment will be clearer and may provide new adjuvant treatment in addition to photocoagulation and laser treatment of diabetic retinopathy.

These articles, hopefully, will provide better understanding of this process and bring forward new ideas with respect to the development of newer adjuvant treatment modalities. However, as evident from these articles as well as data in the literature, the pathogenetic mechanisms involved in diabetic retinopathy are interactive and interdependent. Hence, effective future treatment strategies may include multiple pharmacological agents, targeted simultaneously to block multiple pathways.

Obviously not all aspects of this field could be addressed in one issue and I extend my apologies to many contributors of this field whose work has not been covered. I thank all the authors who contributed to this issue and the reviewers for the highly constructive and helpful comments. My sincere thanks to Dr. A. A. F. Sima for providing me with an opportunity to work as an editor of this issue. It has been my distinct privilege to work with these excellent scientists.

*Subrata Chakrabarti* *Subrata Chakrabarti*

